# Perinatal Antibiotic Exposure and Respiratory Outcomes in Children Born Preterm

**DOI:** 10.1001/jamanetworkopen.2025.9647

**Published:** 2025-05-12

**Authors:** Ingmar Fortmann, Amrei Welp, Nele Hoffmann, Kirstin Faust, Christine Silwedel, Jana Retzmann, Michael Gembicki, Natascha Köstlin-Gille, Anna Häfke, Michael Zemlin, Janina Marissen, Verena Bossung, Janina Soler Wenglein, Jan-Lennard Scharf, Jan Weichert, Andreas Müller, Isabell Ricklefs, Achim Rody, Sabine Pirr, Sebastien Boutin, Jan Rupp, Folke Brinkmann, Martin Heideking, Guido Stichtenoth, Wolfgang Göpel, Egbert Herting, Kathrin Hanke, Christoph Härtel

**Affiliations:** 1 Department of Pediatrics, University Hospital of Schleswig-Holstein, Campus Lübeck, Lübeck, Germany; 2 German Center for Infection Research, Partner Site Hamburg-Lübeck-Borstel-Riems, Lübeck and Borstel, Germany; 3 Department of Gynecology and Obstetrics, University Hospital of Schleswig-Holstein, Campus Lübeck, Lübeck, Germany; 4 Department of Pediatrics, University Hospital of Würzburg, Würzburg, Germany; 5 Department of Pediatrics, University Hospital of Tübingen, Tübingen, Germany; 6 Department of Pediatrics, University Hospital of Saarland, Homburg, Germany; 7 Department of Obstetrics, University Hospital Zürich, Zürich, Switzerland; 8 Department of Pediatrics, Protestant Hospital of the Bethel Foundation, University Medical Center OWL, Bielefeld University, Bielefeld, Germany; 9 Medical School, Bielefeld University, Bielefeld, Germany; 10 Department of Human Medicine, Faculty of Medicine, Witten/Herdecke University, Witten, Germany; 11 Department of Pediatrics, University Hospital of Bonn, Bonn, Germany; 12 Airway Research Center North (ARCN), German Center for Lung Research (DZL), Lübeck, Germany; 13 Department of Pediatric Pneumology, Allergology and Neonatology, Hannover Medical School, Hannover, Germany; 14 Institute of Medical Microbiology, University of Lübeck and University Hospital of Schleswig-Holstein, Campus Lübeck, Lübeck, Germany; 15 Infectious Disease Clinic, University of Lübeck and University Hospital of Schleswig-Holstein, Campus Lübeck, Lübeck, Germany

## Abstract

**Importance:**

Animal models suggest a link between early antibiotic exposure and obstructive airway disease, but corresponding data for premature infants are lacking.

**Objective:**

To investigate whether repeated perinatal antibiotic exposure in preterm neonates with very low birth weight (VLBW) is associated with obstructive airway disease at early school age.

**Design, Setting, and Participants:**

In this population-based, multicenter cohort study, VLBW preterm neonates (22 weeks 0 days’ to 36 weeks 6 days’ gestation with birth weight <1500 g) were enrolled in 58 German Neonatal Network (GNN) centers from January 2009 to March 2017 and received a standardized follow-up at 5 to 7 years of age. To assess the sequential outcomes of antibiotic exposures, the post hoc analysis was restricted to participants born by cesarean delivery. Data were analyzed from May 2024 to February 2025.

**Exposure:**

Perinatal antibiotic exposure, defined by an antibiotic risk score (ARS).

**Main Outcome and Measures:**

The primary end point was the forced expiratory volume in 1 second (FEV_1_) *z* score at 5 to 7 years of age. The low-risk (ARS I) group was exclusively exposed to surgical antimicrobial prophylaxis (SAP) given to the mother before cesarean delivery. The intermediate-risk (ARS II) group was exposed to maternal SAP and postnatal antibiotic treatment of the neonate, while the high-risk (ARS III) group was additionally exposed to antenatal maternal treatment. Secondary outcomes included forced vital capacity (FVC) *z* score and childhood asthma episodes. Univariate and linear regression models were used to analyze outcome measures.

**Results:**

Of 3820 VLBW preterm-born children with follow-up at age 5 to 7 years (median gestational age, 28.4 weeks [IQR, 26.6-30.3 weeks]; 1948 [51.0%] male; 1382 [36.2%] from a multiple birth), 3109 (81.4%) were born by cesarean delivery. Of these children, 292 (9.4%) were classified into ARS I, 1329 (42.7%) into ARS II, and 1488 (47.9%) into ARS III. Higher ARS levels were associated with lower FEV_1_ *z* scores at early school age (ARS II vs I: β, −0.31 [95% CI, −0.59 to −0.02]; *P* = .03; ARS III vs II: β, −0.27 [95% CI, −0.46 to −0.08]; *P* = .006). In the secondary analysis, a higher exposure level (ARS III vs II) was associated with impaired FVC *z* scores (β, −0.23; 95% CI, −0.43 to −0.03; *P* = .02) and an increased risk of early childhood asthma episodes (odds ratio, 1.91; 95% CI, 1.32-2.76; *P* = .001).

**Conclusions and Relevance:**

In this GNN cohort study, multiple episodes of perinatal antibiotic exposure were associated with impaired lung function in preterm-born children at early school age. Early identification of high-risk neonates may enable targeted strategies to support respiratory health and optimize long-term outcomes.

## Key Points

**Question:**

Is repeated perinatal antibiotic exposure in preterm neonates with very low birth weight (VLBW) associated with impaired lung function (forced expiratory volume at 1 second [FEV_1_]) at early school age ?

**Findings:**

In this cohort study of 3820 children born preterm with VLBW, repeated antibiotic exposure during the perinatal and early postnatal period was associated with lower FEV_1_ *z* scores at age 5 to 7 years.

**Meaning:**

These findings suggest that frequent antibiotic exposures during an early critical time frame of neonatal development may have long-term consequences for lung function in children born preterm, highlighting the need for careful antibiotic stewardship in neonatal care.

## Teaser

This cohort study investigates whether perinatal antibiotic exposure in preterm neonates with very low birth weight is associated with impaired lung function at age 5 to 7 years.

## Introduction

Animal models suggest that antibiotic exposure contributes to the development of obstructive airway disease.^1,2,3,4^ Large observational studies of humans have identified associations between prenatal antibiotic exposure and childhood asthma.^5^ Preterm neonates and infants are at increased risk for the development of chronic lung disease compared with their term-born counterparts for many reasons,^6,7^ specifically, preterm lung anatomy, exposure to invasive ventilation and oxygen, and nutritional deficits. The pulmonary outcome of preterm neonates, however, varies greatly given the large number of modifying factors, such as gestational age (GA), genetics, and increased risk of recurrent pulmonary infections.^8^ The increased likelihood of very low-birth-weight (VLBW) neonates later developing chronic obstructive pulmonary disease^9^ and obesity^9,10^ (noncommunicable diseases that have been linked to early-life host-microbiome disturbances) may significantly impact their long-term health risks. Although previous studies have found that antibiotic treatment is associated with the development of asthma in later childhood^11^ in a dose-dependent manner^5,12^ and despite high antibiotic exposure rates in preterm neonates and their increased risk for childhood wheezing, a potential link between early antibiotic exposure and obstructive airway disease has not yet been investigated in this vulnerable population to our knowledge.

Preterm neonates may be exposed to antibiotics at different stages of medical care. First, antenatal exposure occurs when pregnant individuals at imminent preterm delivery receive antibiotic therapies for various reasons, such as premature rupture of membranes, amnion infection syndrome, or any other (suspected) bacterial infection. Nevertheless, antibiotics are often overused in this context,^13^ leading to their use in conditions for which they are not indicated, including preeclampsia; hemolysis, elevated liver enzymes, and low platelet (HELLP) syndrome; or intrauterine growth restriction. Second, surgical antimicrobial prophylaxis for cesarean delivery is recommended in international (including German) guidelines and is routinely administered 30 minutes before delivery.^14,15^ Third, VLBW neonates are often exposed to empirical antibiotics; more than 85% receive antibiotics during the primary stay in German neonatal intensive care units (NICUs) for suspected sepsis.^16,17^ Antibiotics remain lifesaving in the NICU, as delayed or omitted administration can lead to neonatal death or severe organ damage. However, antibiotic exposure enhances the risk for microbiome distortions (gut dysbiosis)—that is, reduction of microbial diversity and key taxa, such as *Bifidobacteria*. In turn, dysbiosis may precede sepsis,^18^ brain injury,^19^ and systemic and sustained inflammation,^20^ all of which contribute to long-term pulmonary morbidities.^21,22^ Due to the lifesaving effect of antibiotics for infections, practitioners in perinatal medicine tend to have a time preference (ie, immediate health concerns are prioritized over potential future risks when determining the indication and duration of antibiotic treatment).^23,24^ In this large-scale German Neonatal Network (GNN) study, we hypothesized that sequential antibiotic exposures during a critical window of mutual microbiome-immune development in VLBW participants would be associated with adverse lung function at early school age.

## Methods

This population-based, multicenter cohort study conducted by the GNN enrolled VLBW neonates at 58 NICUs in Germany from January 2009 to March 2017. The study was approved by the ethics committee of the University of Lübeck and the corresponding ethics committees of the participating hospitals. All participants participated after their parent or guardian gave written informed consent. Our study followed the Strengthening the Reporting of Observational Studies in Epidemiology (STROBE) reporting guideline for cohort studies.

Preterm neonates born between 22 weeks 0 days’ and 36 weeks 6 days’ gestation with a birth weight less than 1500 g who underwent intensive care were included. Data on general neonatal characteristics, antenatal and postnatal treatment, and outcomes were collected for each patient at the participating centers. After discharge, the datasets were sent to the study center (University of Lübeck), coded, and analyzed. A random sample of children participating in the GNN were invited by the study office to a follow-up examination at 5 to 7 years of age. A focus was set on individuals born before 28 weeks’ gestation, but individuals born at more than 28 weeks’ gestation were not necessarily excluded. The study sites were contacted by the study team to arrange follow-up appointments.

**Figure 1.**
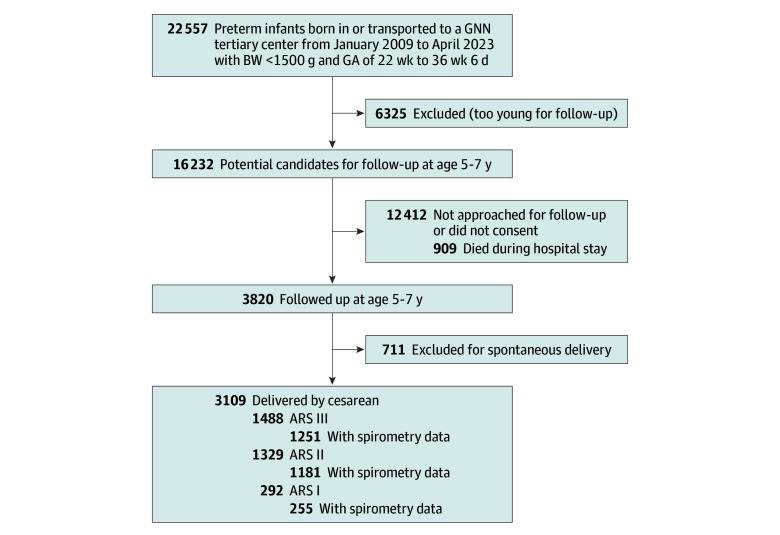
Flowchart of the German Neonatal Network (GNN) Study Cohort of Preterm Individuals With Very Low Birth Weight Included in and Excluded From the Analysis ARS indicates antibiotic risk score; BW, birth weight; GA, gestational age. ARS I indicates 1 antibiotic exposure (lowest risk); ARS II, 2 exposures (intermediate risk); and ARS III, 3 exposures (highest risk).

To ensure the reproducibility of the results, identical instruments and equipment were used for all children and were brought to the study sites by the same GNN team. Visual screening, a hearing test, and spirometry were performed. The parents were given a questionnaire about the children’s medical history, current medical treatments, information about social background, behavior, previous illnesses, and general development. Information on the frequency of bronchitis, wheezing episodes, and need for rehospitalization within the past 12 months was recorded, and these served as secondary respiratory outcomes for this study. For spirometry, an EasyOne spirometer (ndd Medizintechnik AG) was used by the study team physician at all sites. Figure 1 shows the number of children unable to perform spirometry. There were no instructions to the family on changing medication (eg, withholding inhaled medications) before spirometry. The maximum number of spirometry attempts was set to 10. The physician monitored the child’s spirometry performance and assessed the quality by evaluating the volume-time and flow-volume curves of each examination. In addition, an integrated technical validation of volume loops was used. Interactive computer graphics (eg, inflating a balloon) were used to motivate the children to perform spirometry. With reference to European population values^25^ at similar ages, forced expiratory volume in 1 second (FEV_1_) and forced vital capacity (FVC) were documented in liters and analyzed as percentages. The FEV_1_ and FVC *z* scores were calculated based on standard values of the Global Lung Function Initiative.^26^ FEV_1_ of less than 80% was included as a secondary outcome parameter because it was previously established as a predictor for long-term pulmonary morbidity.^27,28,29^

### Definitions

For the analysis of the primary end point, only children born by cesarean delivery with VLBW were included. To stratify children according to an antibiotic risk score (ARS), antenatal antibiotic therapy of the mother (defined as systemic antibiotic therapy within 7 days before delivery), single-dose surgical antimicrobial prophylaxis (SAP) for cesarean delivery, and postnatal antibiotic therapy were considered. Postnatal antibiotic exposure was defined as any exposure to antibiotics for suspected or proven infection during the primary hospital stay.

The ARS was defined by the number of antibiotic exposures: the low-risk group (ARS I) was only exposed to a single dose of maternal SAP prophylaxis 30 minutes before the skin incision. The intermediate-risk group (ARS II) was exposed to SAP and postnatal treatment for suspected infection, and the high-risk group (ARS III) had 3 exposures: antenatal treatment of the mother, SAP, and postnatal antibiotics. Gestational age (GA) was calculated based on early prenatal ultrasonography with measurement of the crown-rump length. Small for GA (SGA) was defined as a birth weight less than the 10th percentile according to VLBW standards.^30^ Moderate or severe bronchopulmonary dysplasia was defined as supplemental oxygen or ventilatory support at a corrected GA of 36 weeks. Bronchitis or wheezing risk was defined by the number of wheezing episodes within 12 months prior to follow-up, as reported in the parental questionnaires.

### Statistical Analysis

SPSS, version 29.0 (IBM Corp) was used for calculations and data analysis. Data were analyzed from May 2024 to February 2025. The type I error level was set to .05. Two-sided *P* < .05 was considered significant. Incomplete datasets were excluded prior to analysis. Analyses were stratified by ARS. A *P* for trend analysis was performed to test for a dose-response relationship between ARS and the primary outcome (FEV_1_ *z* score) using a linear regression model in which ARS was defined as a categorical variable with ordinal levels. To assess the association of antibiotic exposure with the primary outcome (FEV_1_ *z* score), we performed a series of 2 linear regression analyses, treating antibiotic exposure as a categorical variable with 3 exposure levels (ARS I-III). Binary indicators (risk levels 1 and 2) were defined to compare each exposure level with the previous level (risk level 1, ARS II vs I; risk level 2, ARS III vs II). All models, including the *P* for trend analysis, were adjusted for group differences and known perinatal confounding factors affecting pulmonary outcomes of VLBW participants, specifically GA, multiple birth, SGA, anhydramnion, amnion infection syndrome, culture-proven sepsis, bronchopulmonary dysplasia, parental smoking, feeding with own mother’s breast milk, prophylactic treatment with probiotics, mother’s educational level, need for surgery during primary hospital stay on the NICU, exposure to invasive ventilation, duration of invasive ventilation, and risk level 1 or 2.

## Results

### Clinical Characteristics of VLBW Participants Stratified by ARS

From January 2009 to March 2017, a total of 16 232 VLBW participants were enrolled in the 58 GNN centers. The median overall follow-up rate was 23.5% (IQR, 15.9%-27.8%) and varied across participating clinics. A random sample of 3820 children (23.5%) underwent follow-up at 5 to 7 years of age. Of these participants, 1872 (49.0%) were female, 1948 (51.0%) were male, and 1382 (36.2%) were from a multiple birth. Of the 12 412 (76.5%) who were not followed up, 909 (7.3%) had died during the primary stay in hospital. Figure 1 shows detailed information on included and excluded participants. Participants with follow-up were characterized by a lower median GA (28.4 weeks [IQR, 26.6-30.3 weeks] vs 29.0 weeks [IQR, 26.7-30.9 weeks]; *P* < .001) and birth weight (1035 g [IQR, 795-1320 g] vs 1110 g [IQR, 825-1350 g]; *P* < .001) compared with those without follow-up (eTable in Supplement 1). Table 1 shows the clinical characteristics of the 3109 VLBW participants (81.4% of those with follow-up) who were born by cesarean delivery and stratified by ARS: 292 (9.4%) in group I, 1329 (42.7%) in group II, and 1488 (47.9%) in group III. The rate of antenatal antibiotic exposure in VLBW participants born by cesarean delivery was 1488 of 3109 (47.9%), and 2817 (90.6%) were exposed to postnatal antibiotics for suspected or confirmed sepsis. Among these individuals, the median GA was 27.8 weeks (IQR, 26.0-29.9 weeks) and the median birth weight was 970 g (IQR, 740-1230 g). More than one-third of included individuals were from a multiple birth (1349 of 3109 [43.3%]). ARS strata markedly differed in sex predominance (eg, group III had a higher proportion of males), exposure to antenatal steroids, amnion infection syndrome, and SGA.

**Table 1.** Clinical Characteristics and Outcomes of the Follow-Up Cohort Stratified by ARS

Characteristic	Participants^a^		
	ARS I (n = 292)	ARS II (n = 1329)	ARS III (n = 1488)
Gestational age, median (IQR), wk	30.9 (29.4 to 32.3)	28.1 (26.6 to 29.7)	27.4 (25.9 to 29.1)
Birth weight, median (IQR), g	1240 (1058 to 1403)	950 (725 to 1190)	980 (746 to 1220)
Sex			
Female	60.3 (56.7 to 65.3)	49.3 (46.6 to 52.0)	45.6 (43.1 to 48.2)
Male	39.7 (34.2 to 45.4)	50.7 (48.0 to 53.4)	54.4 (51.8 to 56.9)
Multiple birth	36.0 (30.6 to 41.6)	35.3 (32.8 to 37.9)	45.0 (42.5 to 47.6)
SGA	28.4 (23.5 to 33.8)	23.6 (21.4 to 26)	7.7 (6.5 to 9.2)
AIS	1.3 (0.4 to 3.1)	5.6 (4.5 to 7.0)	41.1 (38.9 to 43.9)
Anhydramnion	0.8 (0.1 to 3.7)	1.9 (1.0 to 3.4)	4.8 (3.6 to 7.4)
Antenatal steroids	91.4 (87.9 to 94.2)	87.6 (85.7 to 89.3)	96.6 (95.6 to 97.5)
Bronchopulmonary dysplasia	3.8 (2.0 to 6.4)	23.2 (21.0 to 25.5)	20.2 (18.2 to 22.3)
Intraventricular hemorrhage	2.6 (1.3 to 4.9)	17.5 (15.6 to 19.6)	19.6 (17.6 to 21.6)
Culture-positive sepsis	0	15.3 (13.4 to 17.3)	14.7 (12.9 to 16.5)
ROP requiring intervention^b^	0.3 (0.0 to 1.5)	3.7 (2.8 to 4.8)	4.6 (3.6 to 5.7)
Need for any surgery^c^	6.9 (4.5 to 10.2)	27.1 (24.8 to 29.5)	24.7 (22.5 to 26.9)
NEC requiring surgery	0	3.0 (2.2 to 4.0)	2.3 (1.6 to 3.1)
Parental smoking	37.5 (32.1 to 41.1)	40.5 (37.9 to 43.3)	41.2 (38.7 to 43.8)
Feeding of own mother’s milk	71.2 (63.7 to 77.8)	65.5 (61.8 to 68.9)	68.8 (65.4 to 71.9)
Feeding of donor milk	0.6 (0.1 to 3.0)	4.5 (3.1 to 6.3)	2.5 (1.6 to 3.8)
High school diploma (mother)			
% (95% CI)	49.7 (44.0 to 55.7)	46.4 (43.7 to 49.2)	49.6 (47.0 to 52.3)
No./total No. (%)	144/290 (49.7)	578/1245 (46.4)	681/1372 (49.6)
Rehospitalization in month 12-24			
Mean (SD), d	7.8 (22.4)	11.0 (31.1)	8.11 (28.8)
No. (%)	105 (36.0)	484 (36.4)	541 (36.4)
Hospitalized within 1 y before follow-up			
% (95% CI)	9.1 (6.1 to 12.9)	13.2 (11.4 to 15.2)	12.5 (10.8 to 14.3)
No./total No. (%)	26/285 (9.1)	164/1241 (13.2)	171/1367 (12.5)
FEV_1_			
No. (%)	255 (87.3)	1181 (88.9)	1251 (84.1)
*z* Score, median (IQR)	−1.0 (−1.77 to −0.32)	−1.37 (−2.17 to −0.62)	−1.45 (−2.43 to −0.64)
Volume, median (IQR), L	1.0 (0.87 to 1.13)	0.94 (0.82 to 1.14)	0.93 (0.76 to 1.06)
<80% of Target	16.5 (12.3 to 21.4)	25.9 (23.3 to 28.6)	28.6 (26.1 to 31.3)
FVC			
No. (%)	255 (87.3)	1181 (88.9)	1251 (84.1)
*z* Score, median (IQR)	−1.11 (−1.95 to −0.30)	−1.40 (−2.39 to −0.71)	−1.46 (−2.40 to −0.69)
Volume, median (IQR), L	1.08 (0.92 to 1.23)	1.01 (0.84 to 1.18)	1.0 (0.83 to 1.12)
Asthma episodes 1 y before follow-up			
% (95% CI)	18.9 (14.7 to 23.6)	24.6 (22.3 to 27.0)	26.2 (23.9 to 28.5)
No./total No. (%)	56/295 (19.0)	312/1270 (24.6)	375/1434 (26.2)

Abbreviations: AIS, amnion infection syndrome; ARS, antibiotic risk score; FEV_1_, forced expiratory volume in 1 second; FVC, forced vital capacity; NEC, necrotizing enterocolitis; ROP, retinopathy of prematurity; SGA, small for gestational age.

^a^ Data are presented as percentage (95% CI) of participants unless otherwise indicated. ARS I indicates 1 perinatal antibiotic exposure; ARS II, 2 exposures; and ARS III, 3 exposures.

^b^ Includes laser therapy, cryotherapy, or intraocular administration of vascular endothelial growth factor inhibitors.

^c^ Includes surgery for NEC, focal intestinal perforation, ROP, patent ductus arteriosus, and ventriculoperitoneal shunt.

There were 415 cases of missing lung function data (37 [8.9%] in ARS I, 148 [35.7%] in ARS II, and 230 [55.4%] in ARS III). The most frequent reasons for missing lung function data were the COVID-19 pandemic, during which the performance of spirometry was not allowed at follow-up (192 cases [46.3%]); inability to perform spirometry due to cognitive impairment of the participating child (93 cases [22.4%]); and physical limitations (65 cases [15.7%]), such as cerebral palsy (33 [8.0%]), trisomy 21 (7 [1.7%]), vision impairment (5 [1.2%]), autism (5 [1.2%]), reduced physical resilience due to severe bronchopulmonary dysplasia (5 [1.2%]), severe tracheomalacia (2 [0.5%]), muscular hypotonia (2 [0.5%]), recurrent laryngeal nerve paralysis (1 [0.2%]), Rubinstein-Taybi syndrome (1 [0.2%]), recent tongue or mouth surgery (2 [0.5%]), or unknown underlying disease (2 [0.5%]). Thirty-five children (8.4%) did not cooperate with performing the test, 20 tests (4.8%) were invalid according to the judgment of the follow-up physician, 5 children (1.2%) were not able to participate because of an upper airway infection, and in 5 cases (1.2%), technical problems occurred.

### Association of Antibiotic Exposure With FEV_1_ *z* Scores

**Figure 2.**
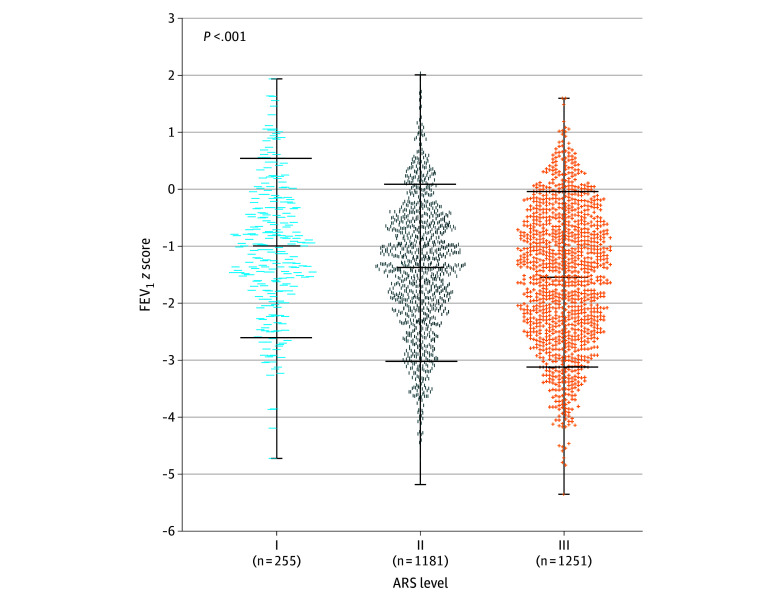
*z* Scores for Forced Expiratory Volume in 1 Second (FEV_1_) Across the 3 Risk Strata of the Antibiotic Risk Score (ARS) Each dot represents an individual data point (*z* score); middle horizontal lines, means; upper and lower horizontal lines flanking the data clouds, 10th and 90th percentiles; whiskers, minimum and maximum values. Statistical comparison among groups was performed using the Kruskal-Wallis test. ARS I indicates 1 antibiotic exposure; II, 2 exposures; and III, 3 exposures.

We observed a significant decline in FEV_1_ *z* scores (primary outcome) associated with increasing ARS levels (Table 1 and Figure 2). A dose-response relationship across the 3 risk groups for antibiotic exposure was confirmed by an adjusted *P* for trend analysis, which demonstrated a significant association between higher antibiotic exposure (ARS I-III) and lower FEV_1_ *z* scores (β, −0.27; 95% CI, −0.40 to −0.13; *P* < .001). This association was further confirmed by linear regression models (Table 2), in which each incremental increase in the ARS was associated with a greater risk of lower FEV_1_ *z* scores. Specifically, compared with ARS I, ARS II (risk level 1) was associated with a mean decrease (β, −0.31; 95% CI, −0.59 to −0.02; *P* = .03), as was ARS III vs II (risk level 2) (β, −0.27; 95% CI: −0.46 to −0.08; *P* = .006).

**Table 2.** Association of Antibiotic Exposure With FEV_1_ *z* Score^a^

Variable	β (95% CI)	*P* value
Gestational age	0.05 (−0.00 to 0.09)	.050
Multiple birth	−0.01 (−0.18 to 0.17)	.94
SGA	−0.26 (−0.67 to −0.10)	.01
Anhydramnion	0.17 (−0.18 to 0.50)	.32
Amnion infection syndrome	−0.30 (−0.53 to −0.08)	.009
Culture-positive sepsis	0.006 (−0.24 to 0.25)	.96
Bronchopulmonary dysplasia	−0.49 (−0.75 to −0.23)	<.001
Parental smoking	−0.10 (−0.09 to 0.29)	.31
Own mother’s milk feeding	0.07 (−0.12 to 0.25)	.49
Probiotics	−0.01 (−0.23 to 0.20)	.19
Mother’s educational level	0.001 (−0.002 to 0.004)	.54
Need for surgery	−0.14 (−0.37 to 0.10)	.25
Invasive ventilation	−0.02 (−0.22 to 0.20)	.88
Duration of invasive ventilation	−0.01 (−0.02 to −0.002)	.02
Risk level 1 (ARS II vs I)^b^	−0.31 (−0.59 to −0.02)	.03
Risk level 2 (ARS III vs II)^b^	−0.27 (−0.46 to −0.08)	.006

Abbreviations: ARS, antibiotic risk score; FEV_1_, forced expiratory volume within 1 second; SGA, small for gestational age.

^a^ *z* Scores were adjusted for sex and postnatal age. All variables included in the regression model are given in the table. *R^2^* was 0.167 for model 1 (or risk level 1), 0.147 for model 2 (or risk level 2), and 0.11 in the model without ARS as an explanatory variable.

^b^ ARS I indicates 1 perinatal antibiotic exposure; ARS II, 2 exposures; and ARS III, 3 exposures.

### Secondary Outcomes

Sequential antibiotic exposure was associated with reduced FVC (association of risk level 2 with FVC *z* score: β, −0.23; 95% CI, −0.43 to −0.03; *P* = .02), increased risk of impaired FEV_1_ (<80% of the target value) (risk level 1: odds ratio [OR], 3.40 [95% CI, 1.07-10.90]; *P* = .04; risk level 2: OR, 1.88 [95% CI, 1.17-3.03]; *P* = .009), and exacerbations of early childhood asthma in the year before follow-up (risk level 2: OR, 1.91 [95% CI, 1.32-2.76]; *P* = .001) (Table 3).

**Table 3.** Association of Antibiotic Exposure With Secondary Outcomes^a^

Secondary outcome	Estimate^b^	*P* value
FVC *z* score, β (95% CI)		
Risk level 1	−0.23 (−0.50 to 0.07)	.14
Risk level 2	−0.23 (−0.43 to −0.03)	.02
FEV_1_ in L, β (95% CI)		
Risk level 1	−0.02 (−0.07 to −0.03)	.40
Risk level 2	−0.04 (−0.08 to −0.01)	.03
FCV, β (95% CI)		
Risk level 1	−0.01 (−0.07 to 0.05)	.83
Risk level 2	−0.04 (−0.08 to −0.002)	.04
FEV_1_<80% of target, OR (95% CI)		
Risk level 1	3.40 (1.07 to 10.90)	.04
Risk level 2	1.88 (1.17 to 3.03)	.009
Asthma episodes 1 y before follow-up, OR (95% CI)		
Risk level 1	1.09 (0.59 to 2.04)	.78
Risk level 2	1.91 (1.32 to 2.76)	.001

Abbreviations: FEV_1_, forced expiratory volume within 1 second; FVC, forced vital capacity; OR, odds ratio.

^a^ Antibiotic exposure was treated as a categorical variable with 3 levels (1, 2, or 3 exposures). Binary indicators were defined to compare consecutive exposure levels (risk level 1, 2 exposures vs 1; risk level 2, 3 exposures vs 2).

^b^ Models were adjusted for the variables shown in Table 2.

## Discussion

In this large population-based cohort of VLBW participants at risk for pulmonary morbidity, we observed an association between sequential antibiotic exposures during the perinatal period and lower FEV_1_ *z* scores at early school age. Although we could not demonstrate a causal link, our data can make a case for balancing the priorities of immediate health concerns of invasive infection and potential long-term consequences when clinicians determine the need for antibiotic treatment.^23^

Preterm birth is frequently mediated by inflammatory conditions, which have been proven to affect lung function in preterm animal models.^31^ In addition, the treatment of an inflammatory condition may affect the delicate microbiome-immune interaction, with consequences for the manifestation of obstructive airway disease.^1,4,32^ In this context, the antibiotic exposure in preterm neonates during a critical window of developmental trajectories is manifold. For example, 80% to 90% of VLBW neonates are born by cesarean delivery^33^ and exposed to at least a single dose of antibiotics for wound infection prophylaxis.^14,15,34^ Antibiotics given to the mother 30 minutes before skin incision are detected in relevant levels in cord blood and may affect the microbiome establishment during the first weeks of life.^35,36^ Almost half of our study’s cohort (47.9%) was exposed to antibiotics through antenatal treatment of their mother within 7 days before preterm birth, while 90.6% of the cohort received postnatal treatment for suspected or confirmed infection. Based on this knowledge, structured programs have been implemented in NICUs to promote a rational use of antibiotics and to avoid overuse.^13,37^ These structured antibiotic stewardship programs have been associated with significantly reduced antibiotic administration rates in NICUs,^38^ while it is still unclear whether effects are sustainable and lead to improved outcomes for neonates and infants. Similar programs and strategies have been established to reduce unnecessary antibiotic treatment during pregnancy and intrapartum.^39,40^ Given the high exposure rates and the urgently indicated use of antibiotics in vulnerable populations, postexposure strategies to stabilize the gut microbiome are crucial. Interestingly, nutrition with human breast milk is associated with improved respiratory outcomes along with protection against infectious complications^41,42,43^ by promoting a *Bifidobacteria*-dominated gut microbiome^44^ and a higher abundance of anti-inflammatory short-chain fatty acids supporting the tolerance to beneficial commensal bacteria and innocuous antigens.^45^

Perinatal antibiotic exposure and consecutive systemic inflammation may increase the risk of an asthma phenotype, which has been associated with microbiome alterations due to cesarean delivery, infections, antibiotic exposure, sustained inflammation, or a combination of these risk factors.^46,47,48^ Hence, in this study, we hypothesized that the risk of lung function impairment would increase with the number of antibiotic exposures. To our knowledge, this is the first study to investigate this association in a cohort of preterm individuals, for whom this issue is particularly relevant due to their high antibiotic exposure rates. However, there are various factors that impact lung development in preterm individuals, and our data suggest that lung function in VLBW individuals is generally poorer compared with their counterparts born at 37 weeks’ GA or later, as indicated by *z* score–defined spirometry results. First, premature birth perturbs alveolar and vascular lung development, resulting in structural simplification and increased airway smooth muscle tissue.^49^ Second, complicated courses of preterm birth are associated with risk of lung impairment, with long-term consequences such as prolonged invasive ventilation, systemic infection, bronchopulmonary dysplasia, and sustained inflammation.^20^ Third, it has been recently shown that polygenic risk scores are associated not only with chronic obstructive pulmonary disease in adults but also with obstructive pulmonary disease of prematurity.^8^ Fourth, preterm individuals may have prolonged susceptibility to infections that extends beyond the neonatal period and predisposes them to repetitive and severe airway infections, potentially contributing to long-term respiratory impairment. Both the condition that supported the clinical decision-making to treat with antibiotics and the treatment itself may contribute to pulmonary morbidity.

However, certain risks of long-term respiratory impairment are difficult to avoid or modify, such as certain antibiotic exposure rates, complicated disease trajectories, and genetic risk profiles. Hence, early identification of high-risk groups enables health care professionals to counsel parents on lung-protective strategies. Preventive recommendations may include structured intervention bundles with key components, such as education on asthma triggers, strict avoidance of passive smoking,^50,51^ support of exclusive breastfeeding for at least 6 months,^41^ regular and thorough follow-up, adherence to vaccination schedules to harness trained immunity effects,^52,53,54^ and judicious antibiotic use in outpatient settings to prevent unnecessary prescriptions for viral infections. Beyond these strategies, a gap remains in targeted interventions for preterm individuals between hospital discharge and early school age. Few structured approaches exist to actively support respiratory health during this critical period, such as the Preterm Inhaled Corticosteroid Intervention study,^55^ providing an example of a postdischarge intervention aimed at improving respiratory outcomes in preterm-born children. Future research should introduce elaborated prevention bundles and develop evidence-based programs to bridge this gap and optimize long-term pulmonary health in this vulnerable population.

### Limitations

This study has limitations. We approached all participating sites and focused on follow-up of individuals born at less than 28 weeks’ gestation, and parents responded to our invitation per their convenience. Due to the multicenter study design and logistic constraints, the overall median follow-up rate of 23.5% (IQR, 15.9%-27.8%) varied across participating clinics. Hence, a selection bias cannot be excluded. Furthermore, we lacked information on dosage and length of perinatal antibiotic treatment. This study described associations between a high ARS and impaired lung function and increased asthma risk at early school age. A causal relationship could not be established, and mechanistic studies (ie, on microbiome effects of antibiotic treatment and their consequences for pulmonary morbidity) are required. Infants with more antibiotic courses are more vulnerable and have worse health compared with infants treated with fewer courses, and these clinical differences may partly account for the associations reported. Other confounding factors, such as gut dysbiosis, exposure to drugs and medications, nutrition and growth, severe infections, or unidentified systematic inflammation, may affect a child’s development and respiratory outcomes. As in previous studies of respiratory performance in preterm individuals,^28,29^ our study is limited by a morbidity bias because only children healthy enough to undergo pulmonary function testing were included.

## Conclusions

In this cohort study, multiple exposures to antibiotics in VLBW preterm neonates were associated with impaired lung function and an increased risk of wheezing at early school age. Antibiotic stewardship programs in clinics of perinatal medicine may help to promote a rational use of antibiotics. While some risk factors for respiratory impairment, such as antibiotic exposure, disease complexity, and genetic predisposition, are difficult to modify, early identification of high-risk individuals allows for targeted parental counseling and structured prevention strategies. Evidence-based programs and prevention bundles are needed to support respiratory health and optimize long-term outcomes in the vulnerable group of preterm individuals.

## Notes

Supplement 1. **eTable.** Clinical Characteristics of GNN Cohort Stratified to Inclusion in Follow-Up

Supplement 2.
Data Sharing Statement
