# Supplementary material for: Perinatal Antibiotic Exposure and Respiratory Outcomes in Children Born Preterm
Source: JAMA Netw Open. 2025 May 12;8(5):e259647. doi: 10.1001/jamanetworkopen.2025.9647 (PMC12070239; doi:10.1001/jamanetworkopen.2025.9647)
Supplement: Supplement 1. — eTable. Clinical Characteristics of GNN Cohort Stratified to Inclusion in Follow-Up [file jamanetwopen-e259647-s001.pdf]

Supplementary Online Content

Fortmann I, Welp A, Hoffmann N, et al. Perinatal antibiotic exposure and respiratory outcomes in children born preterm. *JAMA Netw Open*. 2025;8(5):e259647. doi:10.1001/jamanetworkopen.2025.9647

eTable. Clinical Characteristics of GNN Cohort Stratified to Inclusion in Follow-Up

This supplementary material has been provided by the authors to give readers additional information about their work.

© 2025 Fortmann I et al. *JAMA Network Open*.

eTable. Clinical characteristics of GNN cohort stratified to inclusion into follow-up

	**No follow-up** (n = 12,412)	**5-7-year follow-up** (n = 3,820)	**p**	**Total** (n = 16,223)
**Characteristics**	**median (IQR)**			**median IQR**
**Gestational age** (weeks)	29.0 (26.7 – 30.9)	28.4 (26.6 – 30.3)	<0.001#	28.9 (26.7 – 31.7)
**Birth weight** (grams)	1110 (825 – 1350)	1035 (795 – 1320)	<0.001#	1100 (820 – 1345)
	**% (95% CI)**			**% (95% CI)**
**Gender** (male)	50.9 (50.0 – 51.8)	51.0 (49.5 – 52.6)	0.86	50.9 (50.2 - 51.7)
**Inborn**	96.6 (96.3 – 96.9)	97.6 (97.0 – 98.1)	0.004	96.8 (96.5 – 97.1)
**Caesarean section**	91.2 (90.7 – 91.7)	91.0 (90.1 – 92.0)	0.81	91.1 (90.7 – 91.6)
**SGA**	19.5 (18.8 – 20.2)	15.4 (14.3 – 16.6)	<0.001	18.5 (17.9 – 19.1)
**Multiples**	34.1 (33.3 – 34.9)	36.2 (34.7 – 37.8)	0.02	34.6 (33.9 - 35.3)
**Antenatal antibiotic exposure**	48.2 (47.4 – 49.1)	51.4 (49.8 – 53.1)	0.001	48.9 (48.2 – 49.7)
**Postnatal antibiotic exposure**	78.6 (77.9 – 79.3)	87.2 (86.0 – 88.2)	<0.001	80.5 (79.9 – 81.1)

Legend: *SGA, small-for-gestational-age; IQR, interquartile range. P-values were derived from Chi-square test if not otherwise indicated#.*

© 2025 Fortmann I et al. *JAMA Network Open*.
