# Supplementary material for: Perinatal Antibiotic Exposure and Respiratory Outcomes in Children Born Preterm
Source: JAMA Netw Open. 2025 May 12;8(5):e259647. doi: 10.1001/jamanetworkopen.2025.9647 (PMC12070239; doi:10.1001/jamanetworkopen.2025.9647)
Supplement: Supplement 2. — Data Sharing Statement [file jamanetwopen-e259647-s002.pdf]

Data Sharing Statement

Fortmann. Perinatal Antibiotic Exposure and Respiratory Outcomes in Children Born Preterm. *JAMA Netw Open*. Published May 12, 2025. doi:10.1001/jamanetworkopen.2025.9647

Data
Data available: No

Additional Information
Explanation for why data not available: Data will be shared on reasonable request to the corresponding author.
